# Inflammatory Responses Induced by the Monophasic Variant of Salmonella Typhimurium in Pigs Play a Role in the High Shedder Phenotype and Fecal Microbiota Composition

**DOI:** 10.1128/msystems.00852-22

**Published:** 2023-01-11

**Authors:** Florent Kempf, Guido Cordoni, Anne-Marie Chaussé, Rosanna Drumo, Helen Brown, Daniel L. Horton, Frédéric Paboeuf, Martine Denis, Philippe Velge, Roberto La Ragione, Annaëlle Kerouanton

**Affiliations:** a ISP, INRAE, Université François Rabelais de Tours, Nouzilly, France; b Department of Pathology and Infectious Diseases, School of Veterinary Medicine (VSM), University of Surrey, Guildford, Surrey, UK; c ANSES, SPF Pig Production and Experimental Unit, Ploufragan, France; d ANSES, Hygiene and Quality of Poultry and Pig Products Unit, Ploufragan, France; e University of Surrey, Department of Microbial Sciences, School of Biosciences and Medicine, Guildford, Surrey, UK; University of California Davis

**Keywords:** *Salmonella*, pig, gut microbiota, immunity, high shedder, inflammation

## Abstract

Pigs infected with Salmonella may excrete large amounts of Salmonella, increasing the risk of spread of this pathogen in the food chain. Identifying Salmonella high shedder pigs is therefore required to mitigate this risk. We analyzed immune-associated markers and composition of the gut microbiota in specific-pathogen-free pigs presenting different shedding levels after an oral infection with Salmonella. Immune response was studied through total blood cell counts, production of anti-Salmonella antibodies and cytokines, and gene expression quantification. Total Salmonella shedding for each pig was estimated and hierarchical clustering was used to cluster pigs into high, intermediate, and low shedders. Gut microbiota compositions were assessed using 16S rRNA microbial community profiling. Comparisons were made between control and inoculated pigs, then between high and low shedders pigs. Prior to infection, high shedders had similar immunological profiles compared to low shedders. As soon as 1 day postinoculation (dpi), significant differences on the cytokine production level and on the expression level of several host genes related to a proinflammatory response were observed between high and low shedders. Infection with Salmonella induced an early and profound remodeling of the immune response in all pigs, but the intensity of the response was stronger in high shedders. In contrast, low shedders seroconverted earlier than high shedders. Just after induction of the proinflammatory response (at 2 dpi), some taxa of the fecal microbiota were specific to the shedding phenotypes. This was related to the enrichment of several functional pathways related to anaerobic respiration in high shedders. In conclusion, our data show that the immune response to Salmonella modifies the fecal microbiota and subsequently could be responsible for shedding phenotypes. Influencing the gut microbiota and reducing intestinal inflammation could be a strategy for preventing Salmonella high shedding in livestock.

**IMPORTANCE** Salmonellosis remains the most frequent human foodborne zoonosis after campylobacteriosis and pork meat is considered one of the major sources of human foodborne infections. At the farm, host heterogeneity in pig infection is problematic. High Salmonella shedders contribute more significantly to the spread of this foodborne pathogen in the food chain. The identification of predictive biomarkers for high shedders could help to control Salmonella in pigs. The purpose of the present study was to investigate why some pigs become super shedders and others low shedders. We thus investigated the differences in the fecal microbial composition and the immune response in orally infected pigs presenting different Salmonella shedding patterns. Our data show that the proinflammatory response induced by *S.* Typhimurium at 1 dpi could be responsible for the modification of the fecal microbiota composition and functions observed mainly at 2 and 3 dpi and to the low and super shedder phenotypes.

## INTRODUCTION

Host heterogeneity in animal infection is problematic and can contribute to the emergence and spread of foodborne pathogens. A minority of infected animals, the high shedders, also known as super shedders, are considered responsible for the majority of the infections (1, 2). Heterogeneity in Salmonella infection has been described in mice (2, 3), chickens (4), and pigs (5). Salmonella control on farms is extremely important, as Salmonella is a major cause of zoonotic diseases. Indeed, salmonellosis remains the most frequent human foodborne zoonosis after campylobacteriosis in the European Union (1). Pork meat is considered one of the major sources of human foodborne infections (6).

However, controlling Salmonella in the food chain is challenging because of the ability of Salmonella to colonize livestock without causing clinical disease. Pigs are typically asymptomatic healthy carriers of nonhost-restricted Salmonella serotypes (7) with varied levels and durations of fecal Salmonella shedding postinfection in both experimental and on-farm infections (8). High levels of Salmonella detected on carcasses have been positively correlated with both Salmonella shedding by carrier swine and Salmonella prevalence on a farm (9). In addition, the load of Salmonella can increase after transport, confirming that this phase of the production is also a critical point for the control of Salmonella contamination (10). Among serotypes infecting pigs, the monophasic variant of Salmonella Typhimurium is now one of the most prevalent (11–13) with *S.* Derby or *S.* Typhimurium, and its zoonotic importance in human salmonellosis is also highlighted (1, 14).

It has been reported that within a few hours of ingestion, *S.* Typhimurium can be detected in high numbers in the feces (15). Shortly after infection, epithelial barrier damage and inflammation are observed, mainly in the ileal mucosa, as well as a migration of the pathogen to the gut-associated lymphoid tissue via monocyte-derived cells (16). Moreover, 2 days after infection, Salmonella infection has been shown to impact the microbiome diversity at the mucosa (17). These changes in commensal gut microbiota could contribute to the pathogen’s ability to colonize the gut successfully (18) and suggest that 16s rRNA microbial community analysis could be used to shape intervention strategies to mitigate the effects of Salmonella infection and transmission by a prompt identification of high shedders. These results suggest that the manipulation of certain taxa within the porcine intestinal microbial community could increase disease resistance against Salmonella in pigs (18).

Heterogeneity of Salmonella colonization and excretion is a multifactorial process involving the dose, the Salmonella serotype, the feed, and the host genetics (19, 20). However, the host immune status and gut microbiota composition appear to play a crucial role in the variation of Salmonella shedding in the high and low shedder phenotypes (5).

*In vivo* and *in vitro* gene expression studies (21, 22) as well as proteomic studies (23) have shown that an immune response is stimulated when pigs are infected with Salmonella Typhimurium. Detection of Salmonella by immune cell receptors triggers the innate immune response and induces the production of proinflammatory mediators. The release of chemotactic cytokines attracts other immune cells like dendritic or T cells and initiate a Th1 response (24). Different studies have explored the interaction between immune response and the shedding phenotypes. Pigs that shed elevated Salmonella in their feces have higher IFN-γ levels in serum (25). However, it seems difficult to identify immunological markers that could be useful in predicting shedding status. Classical analyses of differentially expressed (DE) genes (19, 26, 27), comparing future high shedders with future low shedders before infection, have difficulty in finding predictive markers. By using gene coexpression analyses, Kommadath et al. (27) were able to identify modules of coexpressed genes that were associated with the shedding phenotypes before infection. Of four of these modules, two were associated with immune response.

To date, very few studies have analyzed both the immune response and the gut microbiota composition in the same experiment (28, 29). Yet, understanding the changes occurring both in the host gut microbiota and the host immune response due to Salmonella infection is of paramount importance with respect to developing a better understanding of Salmonella pathogenesis and disease progression. Moreover, the analysis of these parameters before infection could lead to the identification of predictive biomarkers, which may provide tools to help prevent Salmonella carriage in pigs. The purpose of the present study was to investigate the dynamics between the pig gut microbiota, the immune response, and the levels of Salmonella shedding in orally infected pigs. Depending on the shedding levels, we determined three groups of pigs, high (HS), intermediate (IS) and low (LS) shedders, where zootechnical observations (temperature, growth, and food intake), immune parameters in the blood (total cell counts, level of anti-Salmonella antibodies, level of cytokines, and expression of immune genes), and gut microbiota (feces, ileum and cecum) composition were analyzed.

## RESULTS

### Salmonella shedding and carriage resulting from experimental infection.

All control pigs remained negative for Salmonella throughout the experiment while all the inoculated pigs shed Salmonella from 1 day postinfection (dpi) to the end of the experiment (21 dpi). Salmonella shedding ranged from 1.48 to 9.09 log_10_CFU/g of feces depending on the day and pig. The excretion peak was observed at 2 dpi, with 7.05 ± 1.77 log_10_CFU/g (median). Although all pigs were inoculated with the same dose of Salmonella, we observed different Salmonella excretion patterns.

**FIG 1:**
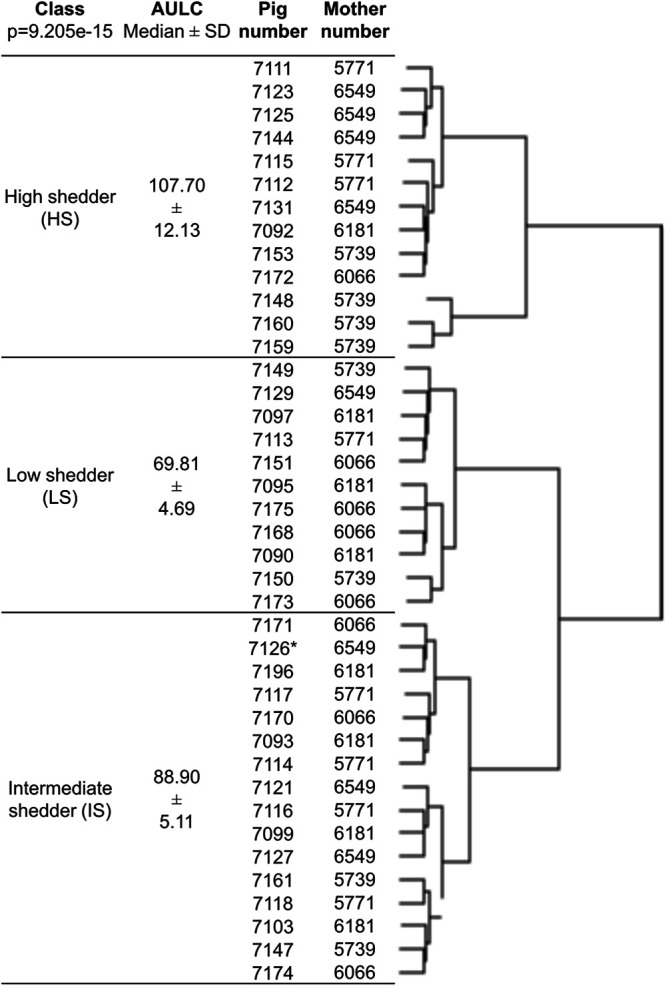
Hierarchical tree produced using AULC data. The analysis classifies the 40 inoculated pigs into three classes (ANOVA, *P* value = 9E-15).

**FIG 2:**
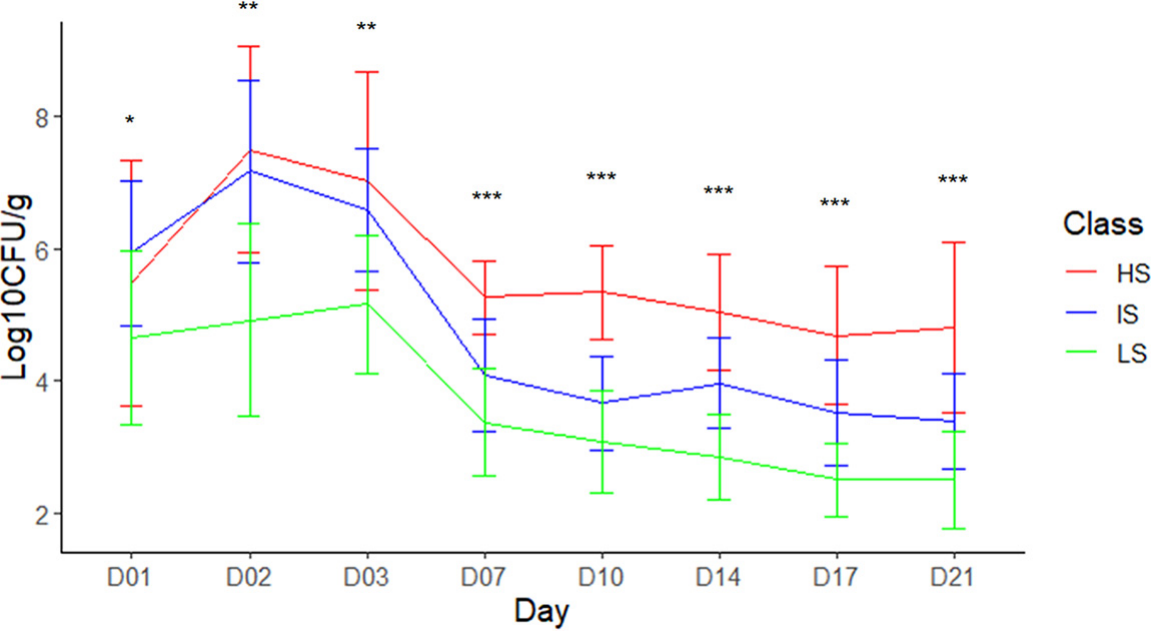
Salmonella enumeration in feces at each sampling day postinfection for each class (median excretion in log_10_ CFU/g ± median absolute deviation according to days postinfection [D01 to D21]). Significant differences found after Kruskal-Wallis comparisons were represented by asterisks (*): the *P* values were *P* = 0.029, 0.001, 0.004, <0.001, <0.001, <0.001, <0.001, and <0.001 at D1, 2, 3, 7, 10, 14, 17, and 21, respectively. Nevertheless, when comparing extreme categories (i.e., HS and LS, Student t-tests), no difference was observed at day 1 (*P* = 0.214), whereas significant differences were observed over the rest of the time series (*P* < 0.001, *P* < 0.001, *P* = 0.012, *P* < 0.001, *P* < 0.001, *P* < 0.001, and *P* < 0.001 at day 2, 3, 7, 10, 14, 17, and 21).

The hierarchical clustering on the area under the log curve (AULC) values permits clustering the pigs into three groups. The three classes (Fig. 1) gathered 13, 16, and 11, high (HS), intermediate (IS), and low shedder (LS) pigs, respectively, with a median AULC of 107.70 ± 12.13, 88.90 ± 5.11, and 69.81 ± 4.69, respectively. These three classes were significantly different (ANOVA, *P* value = 9E-15). For all classes, excretion peak was observed at 2 dpi for the HS and IS class and at 3 dpi for the LS class (Fig. 2).

Pigs from the same sow were found in the three classes, LS, IS, and HS. Furthermore, based on AULC value, there was no difference between pigs according to the sow (*P* value = 0.424) (data not shown), indicating that this parameter has no detectable effect on the excretion level of Salmonella.

**FIG 3:**
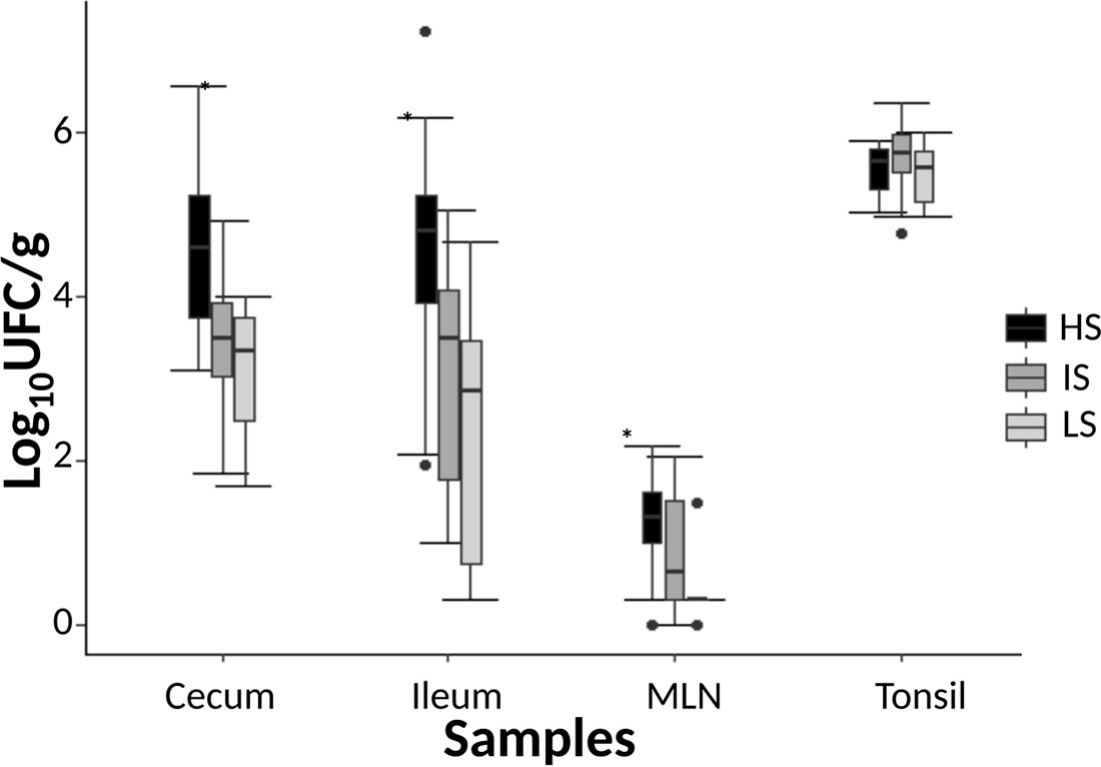
Salmonella enumeration from different sample types, according to excretion classes, at the time of necropsy (D21 to D22 after Salmonella inoculation). Asterisks (*) indicate statistically significant difference between HS and LS classes in the cecum (*P* = 0.002), ileum (*P* = 0.005), and MLN, (*P* = 0.009), but not for the tonsils (*P* = 0.273).

At the end of the experiment, tonsils, cecum, and ileum contents of the pigs were highly contaminated with Salmonella (mean, 5.60 ± 0.37; 3.71 ± 1.06; and 3.46 ± 1.66 log_10_CFU/g, respectively), in contrast to mesenteric lymph nodes (MLN) (mean, 0.86 ± 0.68 log_10_CFU/g). However, for the HS class, levels of Salmonella were significantly higher (*P* value < 0.01) for MLN, ileum, and cecum contents than for the LS group (1.26 ± 0.60; 4.56 ± 1.47; and 4.60 ± 0.96 log_10_CFU/g, respectively, versus 0.35 ± 0.39; 2.45 ± 1.66; and 3.10 ± 0.77 log_10_CFU/g, respectively) (Fig. 3).

### Zootechnical data.

A temperature increase was observed for all inoculated pigs. At 1 dpi, the difference was significant (*P* value < 0.01) between the control group (39.3°C) and inoculated group (39.9°C) but not according to shedding classes HS, IS, and LS.

Growth of the piglets was significantly slower (*P* value<0.005) for HS pigs from 4, 11, and 18 dpi to the end compared to control and LS pigs (Table 1).

**TABLE 1:** Growth in Kg according to time (dpi) and pig classes

Shedding class	4 dpi	11 dpi	18 dpi
HS pigs	16.46+/−2.01	21.28+/−2.32	27.80+/−2.72
LS pigs	19.52+/−3.10	24.60+/−3.59	30.87+/−4.15
Control pigs	19.90+/−2.19	25.10+/−2.05	31.76+/−2.60

### Variation in markers of immunity during the experimental infection.

**(i) Total blood cell counts.** The impact of Salmonella was analyzed on different blood cell populations and leukocyte subpopulations at different time points before infection (data not shown) and postinfection (dpi). Statistical difference in cell numbers between inoculated and control pigs and between HS and LS pigs was evaluated by the Kruskal-Wallis test. The comparison of inoculated pigs to control ones showed that, after inoculation, at some time points, the number of cells between the blood cell populations was different. When compared, the HS and LS showed very few differences. Instead, at 1 and 10 dpi, there was a difference in the number of lymphocytes and in the percentage of granulocytes (Table 2).

**TABLE 2:** List of hematological variables with significant differences between control and inoculated pigs, and between high and low shedder pigs^*a*^

Blood cells	Sampling day	C vs I *P* value	HS vs LS *P* value
White cells			
Leucocytes	1 dpi 7 dpi 10 dpi	**0.030** **0.002** **0.043**	0.120 0.840 0.340
% lymphocytes	1 dpi 7 dpi 10 dpi	**0.001** **0.004** **0.001**	**0.040** 0.170 **0.030**
% basophiles	1 dpi	**0.006**	**0.020**
% eosinophiles	1 dpi	**0.008**	0.320
% monocytes	D0 3 dpi 10 dpi	**0.015** **0.004** **0.049**	0.460 **0.040** 0.210
% granulocytes	1 dpi 7 dpi 10 dpi	**0.001** **0.002** **0.001**	**0.020** 0.090 **0.040**
% other cells	10 dpi 21 dpi	**0.020** **0.014**	0.660 0.330
Red cells			
Red cells	21 dpi	**0.006**	0.560
Haematocrit	21 dpi	**0.010**	**0.040**
Mean cell haemoglobin concn	10 dpi 21 dpi	**0.004** **0.008**	0.200 0.310
Red blood cell distribution index	3 dpi 7 dpi	**0.008** **0.020**	0.884 **0.030**
Platelets	3 dpi	**0.020**	0.310

a Significant differences are shown in bold. C, control; I, inoculated; HS, high shedder; LS, low shedder.

**(ii) Antibody response to Salmonella.** The IDEXX test was used to detect the level of antibodies against Salmonella; an optical density (OD) percentage over 15% indicates that pigs had seroconverted.

**FIG 4:**
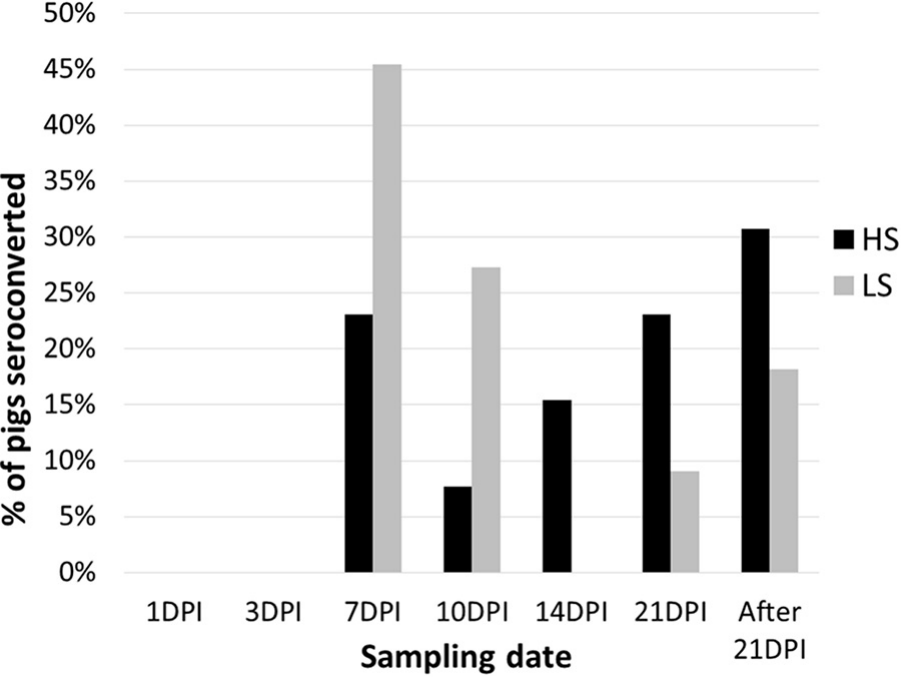
Percentage of pigs seroconverted by classes (HS versus LS) per sampling date.

No seroconversion occurred in control pigs. Inoculated pigs seroconverted from 7 dpi in 12 pigs, from 10 dpi in 7 pigs, from 14 dpi in 4 pigs, and from 21 dpi in 4 pigs. Thirteen pigs did not seroconvert before the end of the experiment, at 21 dpi; 30.80% were HS pigs and 15.40% LS pigs. LS pigs seroconverted earlier than HS pigs in spite of a nonsignificant difference in the level of excretion at 1 dpi between low and super shedders. Seven of the HS pigs (53.80%) seroconverted only at 21 dpi (*n* = 3) or after (*n* = 4) (Fig. 4).

**FIG 5:**
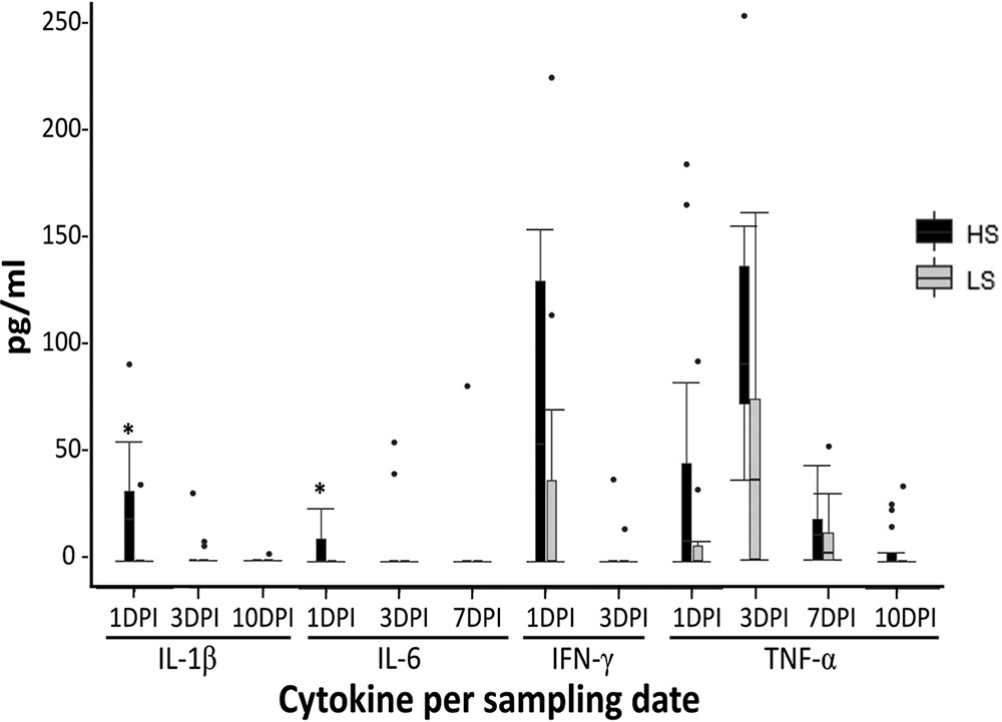
Cytokine production per sampling date. Asterisks (*) indicate the statistically significant difference between classes HS and LS at *P* < 0.05.

**(iii) Cytokine levels.** A peak of production was observed at 1 dpi for interleukins IL-1β, IL-6, and interferon IFN-γ, and at 3 dpi for TNF-α. A significant difference was observed on the median production level between HS and LS for IL-1β and IL-6 at 1 dpi (*P* value = 0.007 and 0.019, respectively) (Fig. 5).

**(iv) Immune gene expression in HS and LS pigs.** To identify biomarkers that could predict the shedding phenotype, we compared immune gene expression between HS and LS pigs before infection. Very few differences were found (Table 3). Twenty days before inoculation, *IL-10* and *CLEC7A* were more expressed in HS pigs while *GNLY*, encoding an antimicrobial protein, a member of the saposin-like protein family that kills various pathogens, including Salmonella, was less expressed. At 7 days before inoculation, *IL-10* was still more expressed in HS pigs, and *CSF3* was less expressed in HS pigs the day of inoculation.

**TABLE 3:** Comparison of gene expression between HS and LS pigs at 20 (D-20) and 7 (D-7) days before infection and the day of inoculation (D0)^*a*^

Sampling day	Gene	HS/LS
D-20	*IL10*	2.3
	*CLEC7A*	2.3
	*GNLY*	−1.8
D-7	*IL10*	3.2
D0	*CSF3*	−2.3

a FC represents the ratio of gene expression in HS pigs compared to LS. Genes with an FC with an absolute value >2, and a *P* value <0.05 are listed.

After inoculation, when HS or LS were compared to noninoculated pigs, out of 70 expressed genes, 46 differentially expressed (DE) genes were found in infected pigs (Table 4). The peak of gene expression modulation was found at 1 dpi for the HS pigs, while the difference in the level of excretion at 1 dpi between low and high shedders was not significant. In this regard, it is interesting to note that the peak production of IL-1β and IL-6, two master cytokines, in the innate immune response, was also found at 1 dpi. In most of cases, the fold changes were higher for HS than for LS pigs; for example, at 1 dpi, *S100A12* and *S100A9* were expressed 49.60 and 12.00 times more in HS than in controls, respectively, but this ratio was only 6.20 and 4.80 in LS. The corresponding proteins bind calcium and zinc and, by regulating several pathways, have proinflammatory properties; this suggests that HS could develop a stronger inflammatory response. Altogether, this indicates that, contrary to what was intuitively anticipated (30), a strong proinflammatory response did not lead to high protection. Expression of 13 genes was decreased in HS pigs compared to the control, whereas no downregulated genes were observed in the LS pigs. The downregulated genes in HS are involved in different branches of immunity. TLR1, TLR6, and TLR3 are receptors that recognize pathogen-associated molecular patterns and trigger cytokine production. TLR1 and TLR6 recognize bacterial acylated lipopeptides, while TLR6 recognizes double-stranded RNA molecules. Expression of *GNLY* was slightly lower in HS than in LS; 20 days before inoculation and 1 day after infection, its expression in HS pigs was downregulated compared to controls. At 7 and 14 dpi, the expression of this gene was higher in HS and in LS compared to controls.

**TABLE 4:** Comparison of gene expression between HS and control pigs or between LS and control pigs at 1, 3, 7, and 14 dpi^*a*^

LS/ control					HS/ control			
1 dpi	3 dpi	7 dpi	14 dpi	Gene	1 dpi	3 dpi	7 dpi	14 dpi
6.20	6.7	2.1		*S100A12*	49.6	20.5	2.1	2.0
16.80				*IDO*	33.0	8.3		
4.90	4.2			*ALOX5AP*	14.4	7.4	2.4	
4.80	6.3			*S100A9*	12.0	12.6	2.4	
4.60	4.2			*CASP4*	10.6	7.9		
3.80	3.4			*ARG2*	9.0	5.7		
4.40	6.6	2.0		*IL15*	8.0	7.6		
4.30	2.9			*TLR4*	6.1	3.3		
3.20	4.0			*IGSF6*	5.8	5.5		
3.10	4.0			*TREM1*	4.9	4.1	2.5	
3.90	2.2			*SOCS3*	4.9	2.3		
	7.6			*MX1*	4.1	11.8	3.6	
2.50	3.4			*CASP1*	3.2	6.0		
				*MYD88*	3.1	2.1		
				*SOCS1*	2.8			
				*CD14*	4.2			
2.00				*SDCBP*	2.1			
2.10				*TNFRSF1A*	2.0			
	3.9			*IL10*		6.6		
	4.2			*MX2*		5.5		
	3.6			*CEBPB*		5.0	2.3	
3.20	3.7			*IL18*		4.4		
				*CLEC7A*		3.9	2.1	
2.50	2.5			*MDA5*		3.5		
	2.5			*INFGR1*		3.2		
	2.2			*EIF2AK2*		3.1	2.2	
				*TLR8*		2.7		
				*IL23A*		2.4		
		2.0		*CASP8*		2.1	2.9	
				*CD86*		2.0		
				*INFAR2*		2.0		
		2.0	3.3	*CCL4*			2.6	3.0
		3.7	5.1	*GNLY*	−4.2		3.7	
				*HSP70*				−2.3
				*HSP60*	−2.0			
				*TGFBR3*	−2.1			
				*TLR6*	−2.1			
				*CSF3*	−2.7			
				*TLR1*	−2.9			
				*GATA3*	−3.2			
				*TNFSF5*	−3.2			
				*SLADQ*	−3.2			
				*SLADR*	−4.2			
				*TBX21*	−4.6			
	−3.2			*TLR3*	−3.2	−2.3		

a FC represents the ratio of gene expression between LS infected pigs compared to controls or between HS infected pigs compared to controls. Genes with an FC with an absolute value >2 and a *P* value <0.05 are listed. Gray cells represent a FC that is higher in HS pigs than in LS.

**FIG 6:**
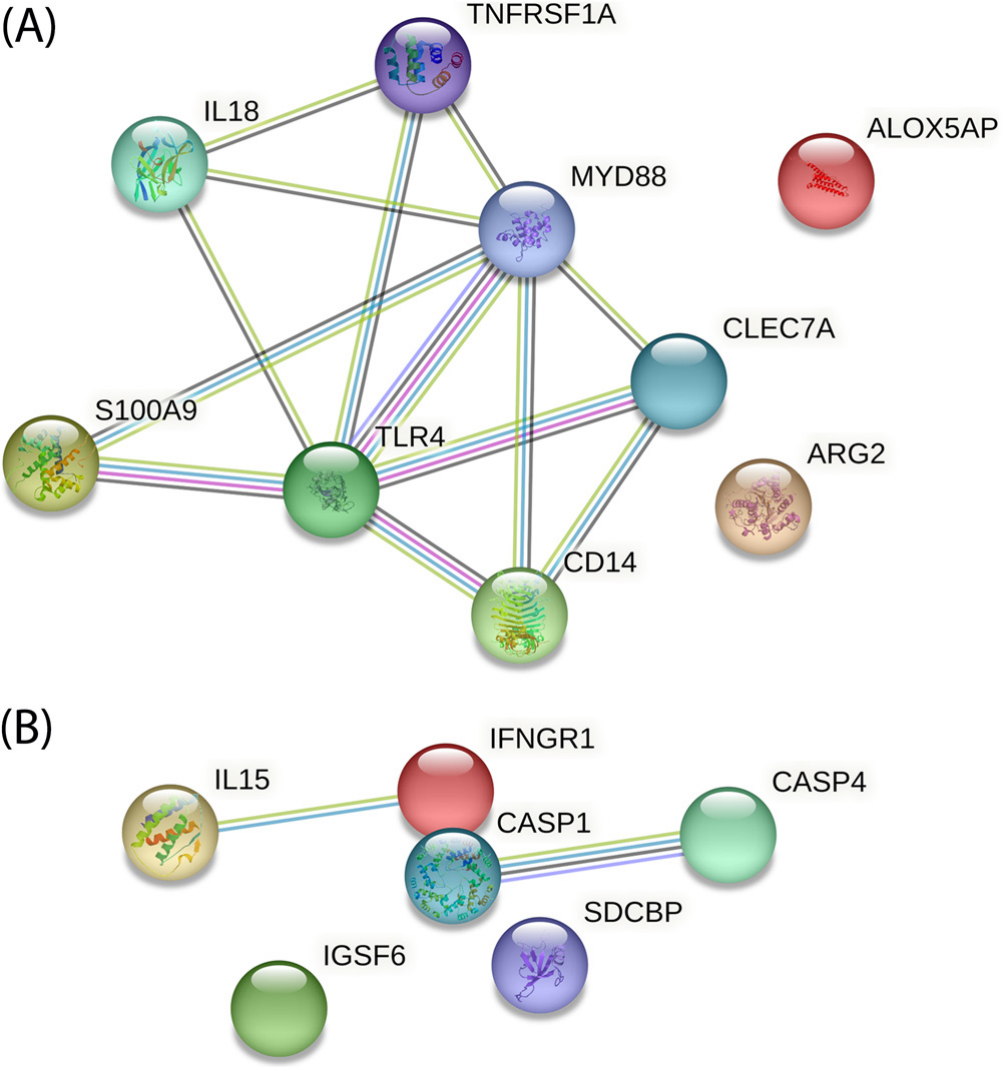
Protein-protein interaction networks corresponding to genes in our experiment that belong to the modules of coexpressed genes associated with shedding phenotype as defined by Kommadath et al. (27). The nodes of the networks correspond to genes and include, when it is known, a 3D representation of the protein. The edges, corresponding to known interaction, are colored according to the source of information: coexpression evidence in black, neighborhood evidence in green, protein homology in pastel blue, cooccurrence evidence in blue, experimental evidence in purple, and database evidence in cyan blue. (A) Pink module. (B) Gray module.

**(v) Correlation with the previously described genes.** Kommadath et al. (27) identified four modules of coexpressed genes which are associated with shedding status, and which are also found before inoculation. Within the four modules the “pink” and the “gray” ones are associated with immune response. In our study, 12 genes belonging to the pink module and 6 to the gray module were upregulated in infected pigs (Table 5). It is assumed that coexpressed genes are involved in common biological processes; we used STRING (31) (https://string-db.org/) to determine how the genes from our study, belonging to the immune modules associated with shedding status, could be functionally linked. Interaction networks between our DE genes, present in the Kommadath’s pink and gray modules, showed that numerous genes of the pink module belong to the same network (Fig. 6A). However, only few DE genes from our study were found in Kommadath’s gray module by using STRING, (Fig. 6B).

**TABLE 5:** List of DE genes in this study that belong to the pink and gray modules of coexpressed genes associated with the shedding phenotype, as defined by Kommadath et al. (27)

Pink module	Grey module
*ALOX5AP*	*CASP1*
*ARG2*	*CASP4*
*CD14*	*IFNGR1*
*CLEC7A*	*IGSF6*
*IL18*	*IL15*
*MYD88*	*SDCBP*
*S100A9*	
*S100A12*	
*TLR4*	
*TNFRSF1A*	

### 16S microbial population analysis in samples collected during and at the end of experimental infection.

**(i) Analysis of fecal samples according to shedding class using the QIIME pipeline.** For these analyses, we considered the fecal samples of all three shedding classes and the control group. The 16S microbial population analysis showed a small but statistically significant difference in bacterial abundance as described below.

**FIG 7:**
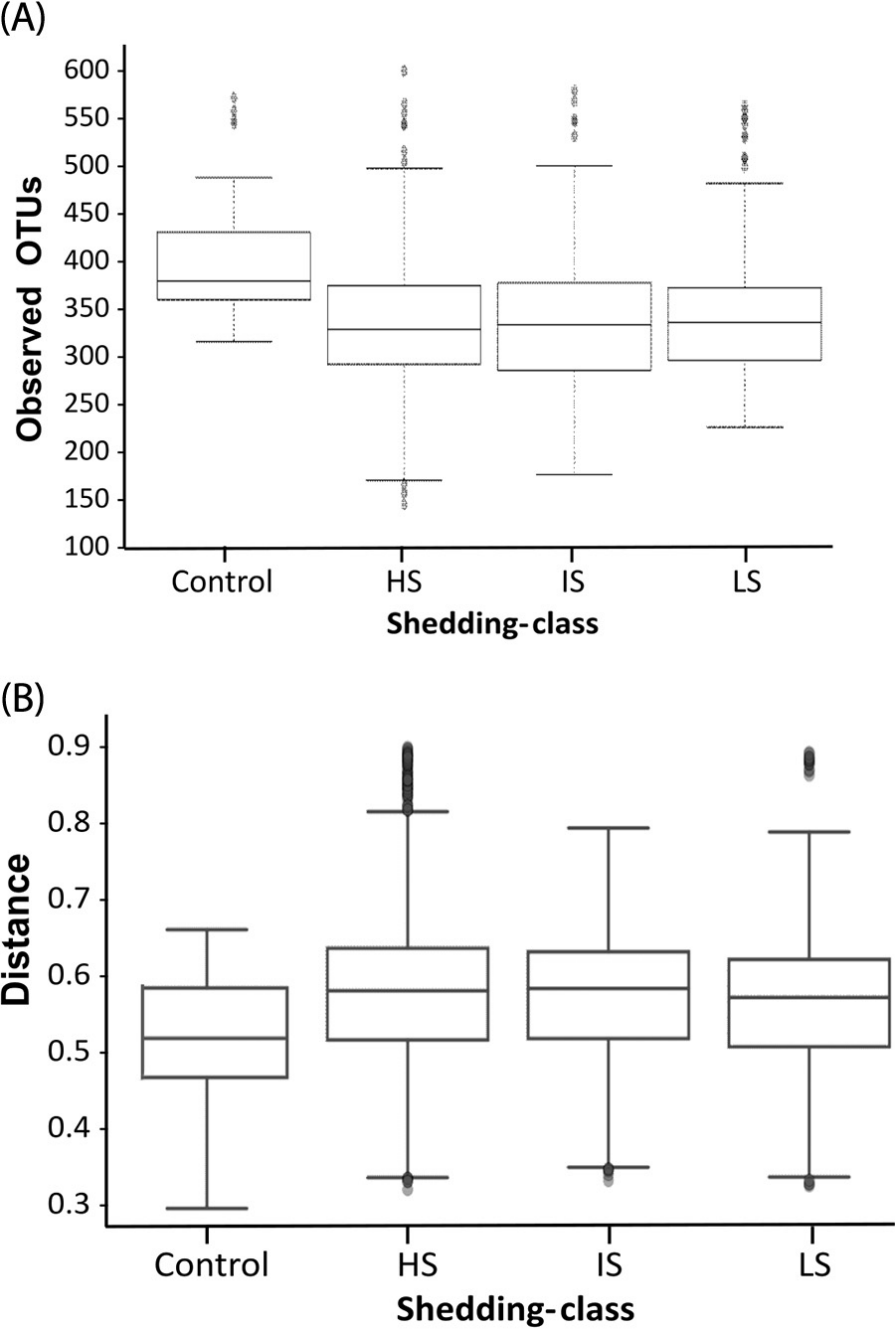
Alpha and beta diversity according to shedding class. (A) Alpha diversity. Fecal samples belonging to the three classes described as HS, IS, and LS show a consistent reduction of the observed OTUs in respect to the control group. No statistically significant differences were detected between HS, IS, and LS with this 16S analysis. (B) Beta diversity (PERMANOVA) class. *P* = 0.001 when controls are compared to HS, IS, or LS; *P* = 0.003 when HS are compared to LS; *P* = 0.018 when HS are compared to IS; *P* = 0.173 for when IS are compared to LS.

Alpha diversity analysis showed statistically significant differences in the number of the operational taxonomic units (OTUs) between each class of inoculated pigs (HS, IS, and LS) and the control group (*P* = 1.55E-04, 8.78E-05, and 1.85E-04, respectively) (Fig. 7A). However, no statistically significant differences were detected in the OTU composition between the three classes defined as HS, IS, and LS. Beta diversity analysis results were similar to those obtained with alpha diversity analysis (Fig. 7B). This result shows that Salmonella colonization modified gut microbiota composition.

**FIG 8:**
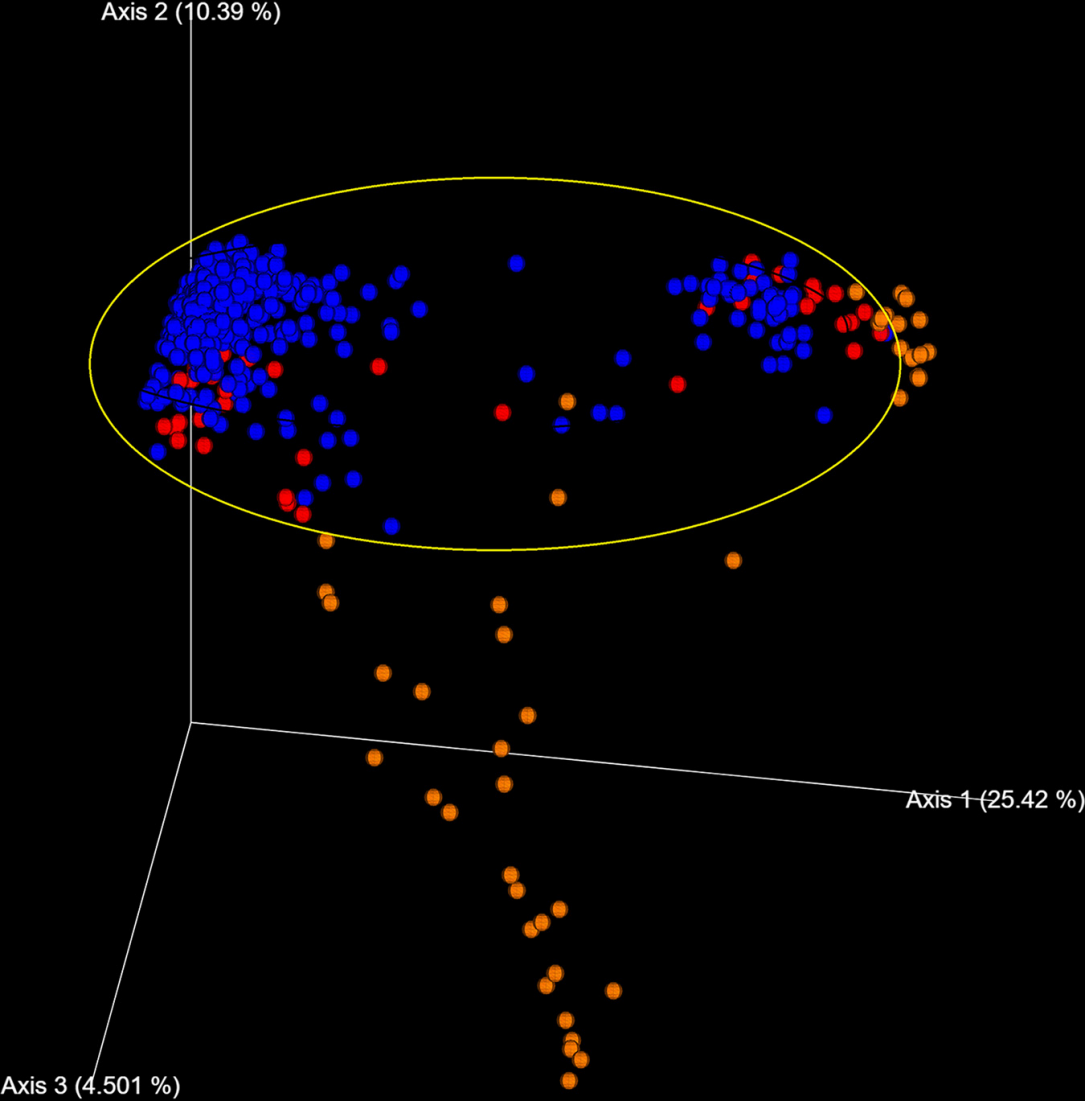
Unweighted UniFrac PCA. PCA was generated from unweighted UniFrac distances. Samples collected from ileum (orange) are clearly separated (an exception was made for few samples) from fecal (blue), and cecal (red) clusters (yellow oval).

**FIG 9:**
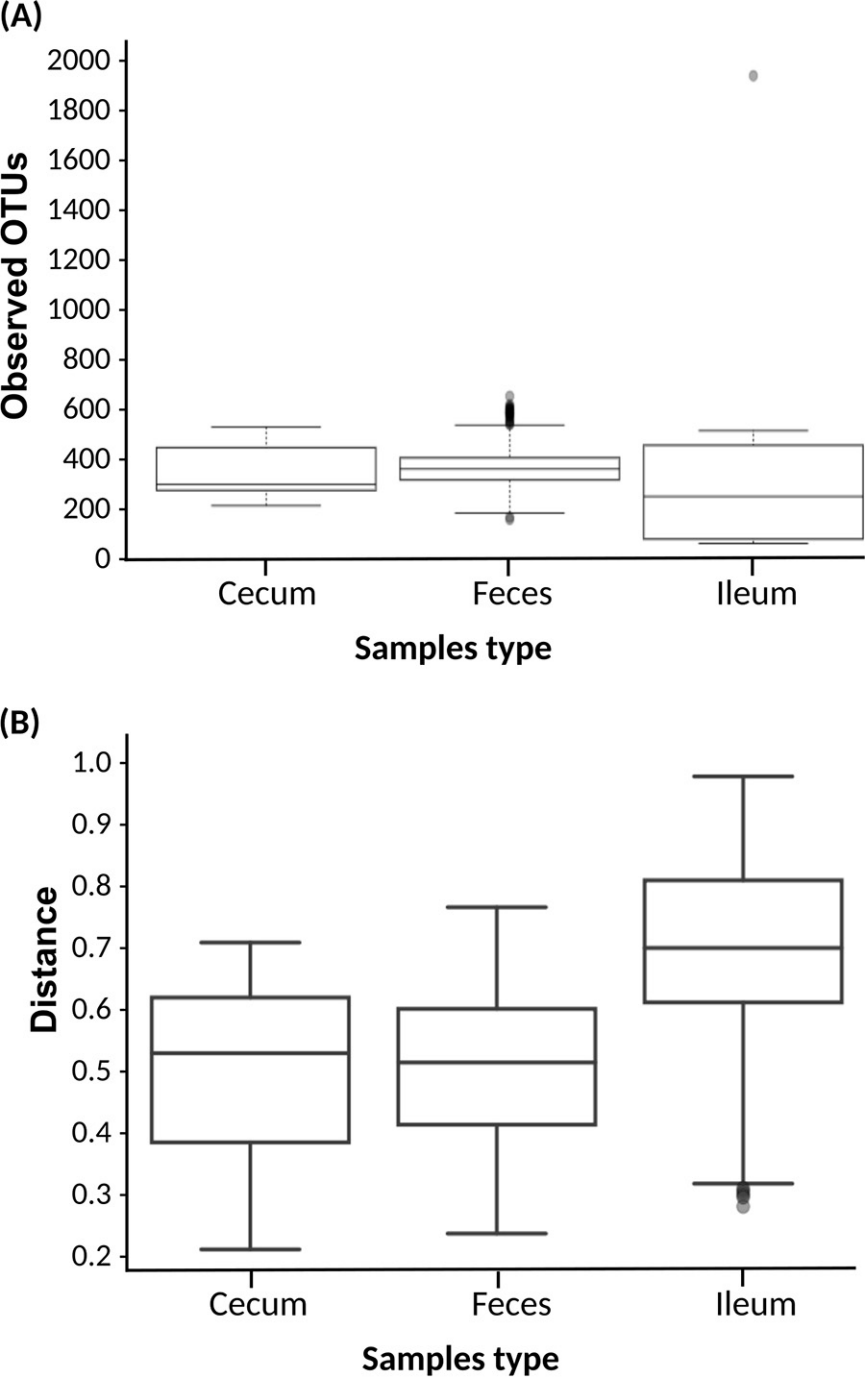
Alpha and beta diversity analysis boxplot according to samples type (feces, cecum, and ileum). (A) Alpha diversity. Small, but statistically significant differences can be noted with respect to feces (H value = 6.174, *P* value = 0.013, q value = 0.039) and ileum (H = 4.103, *P* = 0.043, q-value = 0.086), and the number of observed OTUs in ileum compared with feces (H = 9.081, *P* = 0.003, q = 0.015). (B) Beta diversity. Bacterial composition in ileum shows a statistically significant difference from the bacterial composition in fecal and cecal samples (Table 7).

**(ii) Analysis by sample type (feces, cecum, and ileum) using the QIIME pipeline.** Alpha diversity analysis of infected pigs showed statistically significant differences in the number of OTUs present in the ileum compared to the number of OTUs found in feces and the cecum. This was supported by Kruskal-Wallis (pairwise) tests, where the number of OTUs in cecal samples also showed small but statistically significant differences with respect to feces (H value = 6.174; *P* value = 0.013; q value = 0.039) and ileum (H = 4.103; *P* = 0.043; q = 0.086), and the number of observed OTUs in ileum compared with feces (H = 9.081; *P* = 0.003; q = 0.015). These differences are summarized by the principal component analysis (PCA) of unweighted UniFrac distances shown in Fig. 8 and in the alpha diversity boxplot (Fig. 9A). Beta diversity supported the results obtained with the alpha diversity (Fig. 9B). In particular, the composition and/or relative abundances of bacteria in samples differ according to sample type in infected pigs as well as in control group (data not shown).

**(iii) Other metadata observed.** There were no statistically significant differences in OTU composition of fecal samples according to the mother of origin, or sex.

**(iv) Analysis by age and shedding class (FROGS pipeline).** To go further, we investigated the differences in the fecal microbial composition at each date of sampling and in ileum and cecum microbial composition at the time of necropsy among the three shedding classes (i.e., HS, LS, IS). To consider the heterogeneous and relatively high alpha and beta diversities estimated using QIIME, additional analyses were done using FROGS, a pipeline optimized for cases of heterogeneous abundances and high numbers of species (32).

As observed in the first analysis, the α-diversities did not reveal strong differences except at 2 days after infection (Table 6); the α-diversity was indeed markedly higher for the LS (the mean Chao1 index was 500.8, 452.3, 371.2 for the LS, IS, and HS, respectively; the mean Shannon index was 4.12, 4.00, and 3.85 for the LS, IS, and HS, respectively). This result underlined that, few days after infection, the shedding classes differed for the richness (Chao1) of their gut microbiota. It should be noted that, after removal of the OTUs assigned to Salmonella, the differences of α-diversity remained significant for Chao1 index 2 days after infection (*P* = 0.005).

**TABLE 6:** One-way ANOVA tests comparing the Chao1 and Shannon α-diversity^*a*^

Sampling day and sample type	F value	*P* value
D0 feces		
Chao1	0.107	0.899
Shannon	0.521	0.598
D1 feces		
Chao1	1.949	0.157
Shannon	2.931	0.066
D2 feces		
Chao1	6.467	**0.004**
Shannon	4.810	**0.014**
D3 feces		
Chao1	0.740	0.485
Shannon	1.079	0.352
D7 feces		
Chao1	0.383	0.684
Shannon	1.360	0.270
D10 feces		
Chao1	2.308	0.114
Shannon	2.624	0.086
D14 feces		
Chao1	0.379	0.687
Shannon	1.308	0.283
D17 feces		
Chao1	0.527	0.595
Shannon	0.200	0.819
D21 feces		
Chao1	1.436	0.251
Shannon	0.505	0.608
D22 ileum		
Chao1	0.125	0.883
Shannon	0.133	0.876
D22 cecum		
Chao1	1.133	0.332
Shannon	1.337	0.274

a The comparison among the three shedding categories HS, IS, and LS the day of infection (D0) and at different days postinfection is shown. Significant *P* values (*P* < 0.05) are in bold.

**FIG 10:**
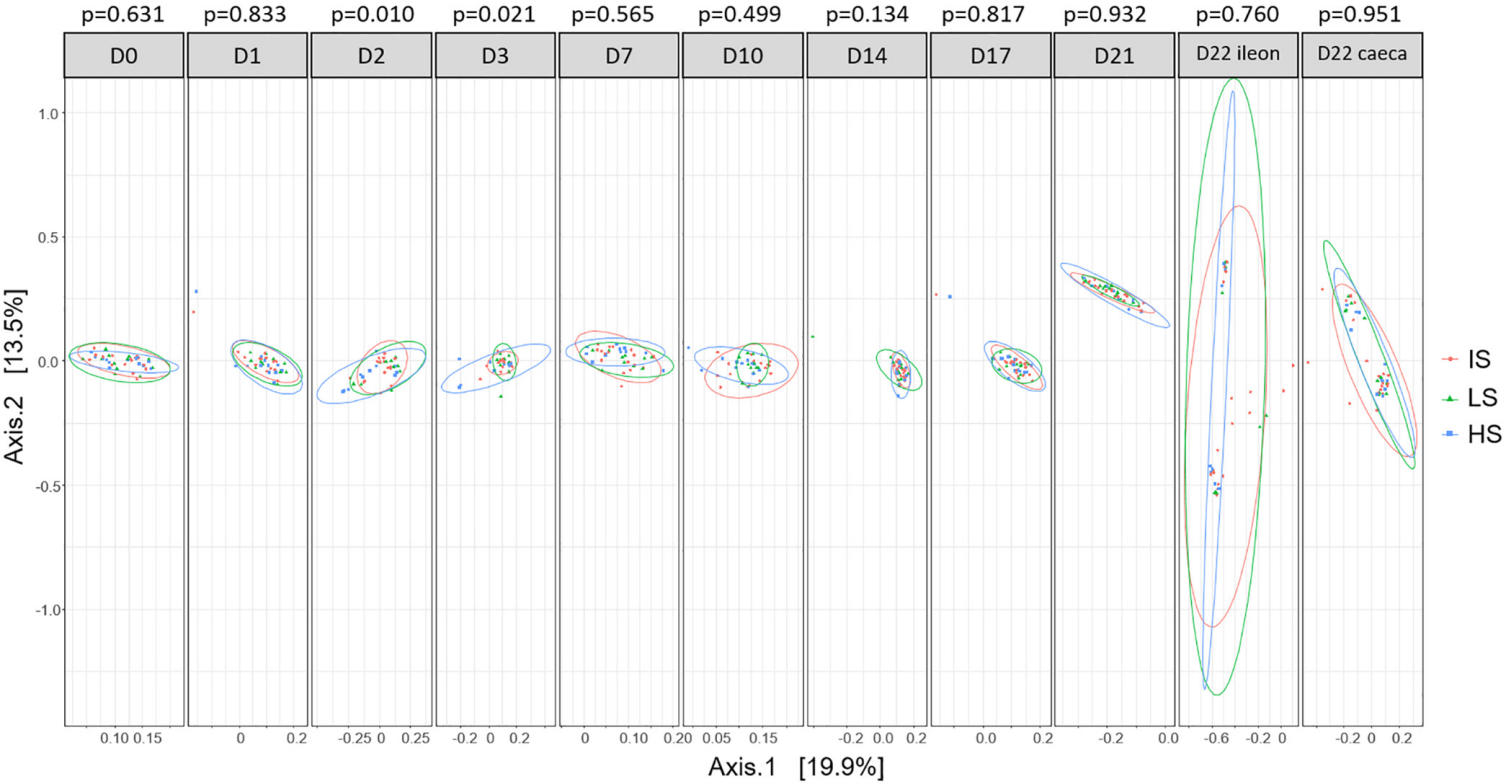
Principal coordinates analyses of the Bray-Curtis β-diversity indexes at every time point. The ellipses represent the distribution of the samples collected in each shedding category, HS (high shedders), IS (intermediate shedders), LS (low shedders). The *P* values of the PERMANOVA comparison of the shedding classes are reported above each plot. From D0 to D21, samples correspond to feces.

The β-diversities (Bray-Curtis index) revealed slight differences among the shedding classes at two and 3 days after infection (*P* value = 0.010 and 0.021, respectively; Fig. 10). In line with this, significant correlations were found by directly fitting bacterial counts on the Bray-Curtis ordination on day 2 and 3 after infection (*P* value = 0.001 and 0.002, respectively). Furthermore, this result was in line with the fact that several OTUs presented differential abundances at these time points (Table 7). The strength of this analysis was supported by the identification of the OTU designated by Cluster_50, which was one of the OTUs assigned to Salmonella, and representing the main part of this genus’ abundance (>95%). According to the bacteriological measurements, this cluster likely corresponded to the inoculated strain. It was found enriched in the HS on day 2 and 3 after infection. Among the other OTUs, whose abundance was significantly different, several facultative anaerobes or aero-tolerant genera were found enriched in the HS (i.e., OTU assigned to the *Bacteroidetes* phylum like *Bacteroides* and *Coprobacter* and to the *Proteobacteria* phylum like *Actinobacillus*, Escherichia and *Desulfuvibrio*). Surprisingly, despite the overall high percentage of *Firmicutes* in the fecal microbiota, only few *Firmicutes*-associated OTU (*Ruminococcaceae*, *Lactobacillaceae*) were significantly different between the HS, IS and LS phenotypes.

**TABLE 7:** OTUs presenting differential abundances in feces among the HS and LS shedding classes^*a*^

Sampling day	OTU	Log_2_ fold change	Phylum	Family	Genus	P.adj
D1	Cluster_61	3.29	*Firmicutes*	*Ruminococcaceae*	[*Eubacterium*] *coprostanoligenes* group	8.49E-03
D2	Cluster_50	–4.37	*Proteobacteria*	*Enterobacteriaceae*	Salmonella	1.64E-04
	Cluster_44	–5.51	*Bacteroidetes*	*Bacteroidaceae*	*Bacteroides*	3.26E-04
	Cluster_145	–5.89	*Fusobacteria*	*Fusobacteriaceae*	*Fusobacterium*	1.92E-06
	Cluster_172	–2.98	*Bacteroidetes*	*Bacteroidaceae*	*Bacteroides*	4.13E-03
	Cluster_423	–6.99	*Proteobacteria*	*Pasteurellaceae*	*Actinobacillus*	2.30E-08
	Cluster_316	–6.34	*Proteobacteria*	*Pasteurellaceae*	*Actinobacillus*	1.55E-07
	Cluster_250	–5.52	*Firmicutes*	*Lachnospiraceae*	[*Acetivibrio*] ethanol gignens group	9.17E-06
	Cluster_18	3.92	*Cyanobacteria*	*Unknown_family*	Unknown genus	7.03E-04
	Cluster_148	–2.77	*Bacteroidetes*	*Marinifilaceae*	*Odoribacter*	3.26E-03
	Cluster_182	–3.28	*Bacteroidetes*	*Barnesiellaceae*	*Coprobacter*	3.78E-03
D3	Cluster_44	–7.80	*Bacteroidetes*	*Bacteroidaceae*	*Bacteroides*	2.21E-03
	Cluster_21	–3.49	*Proteobacteria*	*Enterobacteriaceae*	Escherichia *-Shigella*	2.21E-03
	Cluster_50	–4.72	*Proteobacteria*	*Enterobacteriaceae*	Salmonella	1.26E-03
	Cluster_62	–3.79	*Bacteroidetes*	*Marinifilaceae*	*Butyricimonas*	1.09E-03
	Cluster_145	–12.43	*Fusobacteria*	*Fusobacteriaceae*	*Fusobacterium*	6.81E-04
	Cluster_134	–2.92	*Proteobacteria*	*Desulfovibrionaceae*	*Desulfovibrio*	3.17E-03
	Cluster_172	–3.67	*Bacteroidetes*	*Bacteroidaceae*	*Bacteroides*	2.21E-03
	Cluster_148	–3.60	*Bacteroidetes*	*Bacteroidaceae*	*Odoribacter*	1.07E-03
D10	Cluster_26	–4.09	*Firmicutes*	*Lactobacillaceae*	*Lactobacillus*	1.27E-03

a OTUs enriched in HS and LS are shown in dark gray and light gray cells, respectively. The log_2_ fold change and corresponding adjusted *P* value (P.adj) are reported. Only the most abundant features (N_seq_ > 1,000) are reported.

**(v) Functional enrichment analysis.** The functional enrichment analysis revealed that the main differences among the three shedding classes were observed on day 2, 3, and 17 after infection. However, for clarity, only comparisons between LS and HS are described Table 8. These differences included (i) an enrichment of short-chain fatty acid production in the HS (i.e., pathway noted P162-PWY) and (ii) enrichments of several menaquinol biosynthesis pathways. Menaquinone biosynthesis pathways are essential in electron transport and ATP generation in all Gram-positive, and anaerobically respiring Gram-negative bacteria. This result suggested that anaerobic respiration is induced in HS by using electron acceptors other than molecular oxygen (O_2_) such as nitrate (NO3−), fumarate, sulfate (SO42−), or sulfur (S). These pathways were highly induced but only at 3 dpi (Table 8). On day 17 after infection, similar pathways were detected but mainly from the comparison of the IS and LS with high levels of menaquinol biosynthesis in the IS (data not shown).

**TABLE 8:** MetaCyc pathways presenting differential abundances in feces among the HS and LS shedding classes^*a*^

Sampling day	MetaCyc pathway	Log2 fold change	Description	P.adj
D2	TYRFUMCAT-PWY	−3.10	l-tyrosine degradation I	3.469E-03
	P163-PWY	1.89	l-lysine fermentation to acetate and butanoate	6.136E-03
	P281-PWY	3.27	3-phenylpropanoate degradation	3.060E-03
	PWY-6562	5.12	Norspermidine biosynthesis	5.793E-05
D3	GLYCOCAT-PWY	4.60	Glycogen degradation I (bacterial)	9.416E-03
	P162-PWY	1.84	l-lysine fermentation to acetate and butanoate	9.416E-03
	P163-PWY	1.87	Purine nucleobases degradation I (anaerobic)	6.375E-03
	P281-PWY	2.51	Glycolysis V (Pyrococcus)	6.406E-03
	PPGPPMET-PWY	3.04	ppGpp biosynthesis	1.585E-03
	PWY-5837	2.40	Superpathway of menaquinol-8 biosynthesis I	8.554E-03
	PWY-5838	1.86	Superpathway of menaquinol-7 biosynthesis	9.416E-03
	PWY-5840	1.83	Superpathway of menaquinol-9 biosynthesis	9.42E-03
	PWY-5845	1.99	Superpathway of menaquinol-6 biosynthesis I	9.42E-03
	PWY-5850	1.99	Ubiquinol-7 biosynthesis (prokaryotic)	9.42E-03
	PWY-5860	2.13	Superpathway of demethylmenaquinol-8 biosynthesis	9.42E-03
	PWY-5861	2.05	Superpathway of demethylmenaquinol-9 biosynthesis	9.42E-03
	PWY-5862	2.13	Superpathway of phylloquinol biosynthesis	9.42E-03
	PWY-5863	2.36	Superpathway of menaquinol-10 biosynthesis	8.55E-03
	PWY-5896	1.99	Superpathway of menaquinol-11 biosynthesis	9.42E-03
	PWY-5897	1.93	Superpathway of menaquinol-12 biosynthesis	9.42E-03
	PWY-5898	1.93	Superpathway of menaquinol-13 biosynthesis	9.42E-03
	PWY-5899	1.93	Superpathway of geranylgeranyldiphosphate biosynthesis I (via mevalonate)	9.42E-03
	PWY-7013	1.99	Protein N-glycosylation (bacterial)	1.58E-03
	PWY-7328	1.40	Superpathway of UDP-N-acetylglucosamine-derived O-antigen building blocks biosynthesis	9.43E-03
	THREOCAT-PWY	3.78	Superpathway of l-threonine metabolism	7.81E-04
D7	PWY-6892	5.19	Thiazole biosynthesis I (E. coli)	8.46E-03
D10	P562-PWY	−1.82	Myo-inositol degradation I	3.62E-03
D17	THREOCAT-PWY	2.96	Superpathway of l-threonine metabolism	9.57E-03

a Pathways enriched in HS and LS are written in dark gray and light gray, respectively. The log_2_ fold change and corresponding adjusted *P* value (P.adj) are reported.

## DISCUSSION

In our study, we observed that pigs experimentally infected with the same dose of a monophasic variant of Salmonella Typhimurium exhibited very different shedding levels (differences of more than 7 log) and we could distinguish three groups of shedding, namely, high, intermediate, and low shedders, based on a kinetic analysis. Such different shedding patterns were described in a previous experimental study of *S.* Typhimurium infection in pigs (26), introducing the notions of persistent and low shedding.

In addition, in our study, when pigs were high shedders, they also exhibited a significantly higher level of Salmonella in mesenteric lymph nodes, ileum, and cecum compared to low and intermediate shedders. In line with result, the high shedder phenotype was found to be related to high cecal colonization (4). We also demonstrate that, these different shedding patterns were not linked to the mother or sex because high and low shedder pigs can originate from a same mother and were equally detected in males and females.

As HS have a much higher transmission rate, they constitute a key target for epidemiological investigation and disease management. Consequently, defining the conditions that favor the HS phenotype is a prerequisite to control the reservoir of contamination within a population. Moreover, to lead these interventions, we need to improve our knowledge on markers in gut microbiota (33) and/or in immune response (19, 25, 26) that could promote the high excretion in pigs.

In the first part of this study, we analyzed immunological parameters that could represent a signature of the shedding status and could identify high shedders before infection. It may be intuitively assumed that a less robust immune response could result in a high shedding level after infection (30). Contrary to that, before infection, we found that there was no difference in the level of production of the cytokines IL-1β, IL-6, TNF-α, and IFN-γ between HS and LS pigs. In addition, we analyzed the expression of 70 immune-related genes before infection and we found only very few differences in gene expression between the shedding classes. Surprisingly, among these few genes, the comparison of expression levels in LS and HS indicated that the latter could be less effective in mounting an immune response. Indeed, at 20 and 7 days before inoculation, the *IL-10* gene, encoding a protein with potent antiinflammatory activities, was more expressed in HS pigs. In the same way, at 7 days before infection, expression of *GNLY*, encoding a protein with robust antimicrobial activity against various pathogens, including Salmonella, was slightly less expressed in HS pigs (34). Similarly, *CSF3*, that may reduce Salmonella shedding upon infection in pigs (35), was less expressed in HS the day of infection. On the other hand, early after infection, compared to noninfected pigs, expression of *GNLY* and *CSF3* but also that of *TLR1*, *3*, and *6*, key players of innate response development, was downregulated only in HS pigs (Table 4). Downregulation of expression of these genes could be related to a partial immunosuppressive activity exerted on HS by the invading Salmonella.

In agreement with what was observed in previous studies (26, 36), we found that infected pigs exhibited an increased production of the key proinflammatory cytokines IL-1β, IL-6, and TNF-α and that HS pigs were higher producers of these cytokines compared to LS pigs (26). It is important to note that at 1 dpi, the strong proinflammatory response could not be explained by higher Salmonella colonization, as the HS and LS phenotypes were mainly related to the level of Salmonella after the second day postinfection (Fig. 2). Consistent with other reports, we observed a peak in the production of the Th1-produced cytokine IFN-γ at 1 dpi and HS pigs were again higher producers compared to LS (25, 26). After 3 dpi the, differences between infected and control pigs decreased, showing that the burst of immune response is observed in the first days after infection and correlated with the high level of Salmonella shedding observed from 2 dpi.

In agreement with other studies (19, 26, 27), we found that the peak in the modulation of gene expression was as early as 1 dpi and that HS pigs developed a stronger immune response. Kommadath et al. (27) have described four modules of coexpressed genes associated with the shedding status; two of them, the pink and the gray, are related to immune response. A significant number of our DE genes belonged to these immune-related modules. In the pink module, most of the genes are involved in the activation of the transcriptional activator NF-κB. Recognition of the lipopolysaccharide by TLR4 and CD14 recruits the downstream adaptor component MYD88, leading to the final activation of NF-κB. By fostering the expression of MYD88 and CD14, TREM1 acts as an amplifier of the TLR4 signaling (37). CLEC7A is an innate receptor whose dimerization after recognition of β-glucans leads to recruitment of SYK then to the activation of NF-κB (38). S100A12 and S100A9 may be recognized by the innate receptors RAGE and TLR4; RAGE may activate different signaling pathways also resulting in NF-κB activation. Similarly, recognition of the master proinflammatory cytokine TNF-α by TNFRSF1A, one of its receptors, also induces NF-κB activation.

It is noteworthy that, within the top 10 upregulated genes as early as 1 dpi, we found genes exhibiting an immunosuppressive activity like *IDO*, which mediates regulatory T-cell proliferation and exerts antiproliferative activity on effector T cells (39), and *SOCS3*, an inhibitor of cytokine signaling (40). The increased expression of these four genes, with alternative activities, probably illustrated that in order to prevent the pathological consequences following the triggering of the immune response, infected pigs also trigger an early antiinflammatory response concomitant with the establishment of the proinflammatory one. Nevertheless, the stronger immune response in HS is puzzling because one could have anticipated that the high shedding level was the consequence of a less robust immune response (30). However, different hypotheses could solve this apparent paradox. First, the LS could develop a more efficient or a faster immune response and by the time gene expression was analyzed the immune response had returned to the steady state. However, this seems unlikely because gene expression analyses were done very early after infection, at 1 dpi. Another hypothesis relies on the strong interaction between gut microbiota and immune response. Several studies agree indeed that the local inflammation, resulting from the immune response elicited by Salmonella, triggers changes in the gut environment, which promote the growth of Salmonella and prompt changes in the gut microbiota composition (28, 41). Our data strongly argued for this hypothesis. The fact that the proinflammatory response is observed just before the high level of Salmonella colonization suggested that intestinal infection with *S.* Typhimurium results in inflammation, which induced dysbiosis favoring the HS phenotypes.

This inflammatory response is thought to enable Salmonella to compete with the resident gut microbiota using tetrathionate produced by the oxidation of thiosulfate by reactive oxygen species (ROS) or due to its resistance to defensive molecules such as ROS or lipocalin-2, which is secreted by neutrophils and limits the acquisition of iron (42). This hypothesis could explain why the strong inflammatory response observed in HS is correlated with the highest Salmonella shedding levels (43–45). The analysis of the fecal microbiota composition and of the putative functions of this microbiota supported this last hypothesis.

The 16s rRNA microbial community profiling showed that α-diversity indexes as well as β-diversity differed according to the origin of the sample (ileum content, cecal content, feces) in accordance with previous results (46). When samples were split by sample type, the influence of the infection on the ordination of the microbiota was observed. Salmonella infection significantly modified fecal microbiota composition, as both alpha and beta diversities were modified in the different groups of infected pigs (HS, IS, LS) compared to the control pigs. This result is in line with the data obtained in chickens and pigs (5, 47).

Differences in OTUs suggested that it is possible to distinguish HS, IS, and LS at several time points (i.e., 2 dpi and 3 dpi). This was observed after an analysis performed with FROGS. The overall α-diversity was thus higher in the LS at 2 dpi, which is consistent with the results showing that low diversity is often correlated with dysbiosis and susceptibility to infection. Moreover, these differences arrived just after the proinflammatory burst. Among the OTUs involved in these differences, we observed an enrichment of *Ruminococcaceae* and of *Cyanobacteria* in LS compared to HS. This result is consistent with those of Argüello et al. (17) who noticed that some members of the *Clostridia* were more abundant and there was an overall relative increase in cellulolytic microbiota (*Ruminococcus* and *Prevotella*) in nonshedders and noninfected pigs during the weaning and growing stages. In contrast, numerous other bacterial genera were significantly increased in HS compared to LS, showing a dramatic modification of fecal microbiota composition in HS. Besides one OTU assigned to Salmonella that likely corresponded to the inoculated strain, other *Enterobacteriaceae* such as Escherichia, but also several microaerophilic and aero-tolerant taxa (*Lactobacillaceae*, *Pasteurellaceae*) were more abundant in HS compared to LS. This may reflect higher levels of epithelial oxygenation in the HS, even though several anaerobic bacteria are also more abundant in HS (*Fusobacteria* and *Bacteroidetes* families).

In line with these observations, we found that several functional pathways that may be involved in anaerobic respiration were enriched in the HS. Anaerobic respiration is a metabolic route favoring Salmonella over commensal bacteria, which are unable to use host-derived electron acceptors like ROS (48). For example, at 3 dpi, numerous superpathways related to menaquinol biosynthesis are induced in HS compared to LS. Menaquinone biosynthesis pathways are essential in electron transport and ATP generation in anaerobically respiring Gram-negative bacteria, which have been described as a major way of *Enterobacteriaceae* like Salmonella to overgrow commensal bacteria, which used fermentation (49). Anaerobic respiration may be induced in HS by using oxygen as an electron acceptor. Moreover, electron acceptors other than molecular oxygen (O2), such as nitrate (NO3−), fumarate, sulfate (SO42−), or sulfur (S) can be used. We also observed that glycogen degradation pathway is induced in HS compared to LS. Glycogen metabolism in numerous bacteria can be derived from a common pathway and glycogen reserves enable survival when energy consumption is low. The endogenous glucose polymer, glycogen, appears to play an important role in colonization, since mutants that are unable to synthesize or degrade glycogen have significant colonization defects. In support of the hypothesis that E. coli relies on internal carbon stores to maintain colonization during periods of famine, Jones et al. (50) found that by providing a constant supply of a readily metabolized sugar in the animal's drinking water, the competitive disadvantage of E. coli glycogen metabolism mutants is rescued. In line with this, McMeechan et al. (51) found that the glycogen production plays a role in the survival of Salmonella during intestine colonization. An important implication of these observations is that in HS, the relatively high abundance of *Enterobacteriaceae*, including Salmonella and E. coli, was related to a high level of glycogen use as the primary carbon and energy storage molecule. Two pathways related to amino acid degradation and fermentation, namely, “l-lysine fermentation to acetate and butanoate” and the pathway “superpathway of l-threonine metabolism,” were also enriched in the HS (the second being chimeric and including catabolic pathways). Not all amino acids are equally suitable for fermentation, and differences in bacterial growth are observed when single amino acids are compared as sole energy sources in culture media. The highest growth is observed from catabolism of glutamate, arginine, glycine, serine, phenylalanine, and tyrosine (52). Recently, evidence has emerged that aromatic amino acids (phenylalanine, tyrosine, and tryptophan) can be fermented to phenylpropanoid metabolites, phenylacetic acid, and 4-hydroxyphenyl-acetic acid, which are abundant in feces. The organisms involved include several species of *Bacteroides*, Eubacterium hallii, and *Clostridium barlettii* (53). These observations may explain the enrichment of the two pathways related to amino acid degradation and fermentation, insofar as several OTUs assigned to *Bacteroides* were found enriched in the HS.

In contrast, only few pathways were found enriched in the LS compared to the HS. This included the “tyrosine degradation pathway.” This observation was consistent with lower levels of Salmonella and E. coli in LS, given that this pathway is absent in these two taxa.

In our study, the role of short-chain fatty acid (SCFA) production remained unclear, insofar as we expected to observe low levels of SCFA production in the HS. SCFAs, such as butyrate, serve as a major source of energy for the intestinal enterocytes and signals to the host. Moreover, there is considerable interest in these butyrogens since butyrate reduces proinflammatory signals and has a protective role in maintaining a healthy colon. Butyrogenic gut bacteria belong to *Lachnospiraceae* and *Ruminococcaceae* (54). Consistent with the protective activity of SCFA, *Ruminococcaceae* were more abundant in LS than in HS (Table 7). However, the *Lachnospiraceae* were more abundant in HS than in LS. Moreover, most butyrate produced in the human intestine is assumed to derive from carbohydrates, and in our MetaCyc pathways, only the “l-lysine fermentation to acetate and butanoate” pathway was differentially detected. This point requires further investigation, including direct quantification of SCFA production at every time point in a similar pig model.

### Conclusion.

Overall, this study demonstrated that when pigs are orally inoculated with Salmonella, heterogeneity in Salmonella shedding is observed. Hierarchical clustering on AULC permitted to clustering of pigs into three groups: high (HS), intermediate (IS) and low shedder (LS) pigs. The HS pigs had a higher Salmonella shedding level and a higher level of organ colonization, and they grew significantly slower than the control and LS pigs.

Our analysis of immune cell counts and gut microbiota composition did not allow us to identify clear predictive biomarkers for these shedding phenotypes. Interestingly, we observed a higher Interleukin-10 gene expression in the future HS pigs at two time points before infection. Interleukin 10 is a cytokine with potent antiinflammatory properties that plays a central role in limiting host immune response to pathogens. Salmonella inoculation induces a profound and rapid modification of the immune response, which comes just before the modification of the fecal microbiota composition. The immune response is higher in HS than in LS and therefore does not appear to be a protective response. Furthermore, the strong proinflammatory response observed in gene expression and cytokine production at 1 dpi could be responsible of the high Salmonella levels. This is in line with the results obtained in mouse models where the proinflammatory response tends to provide a competitive advantage to Salmonella compared to other bacteria of the gut microbiota. This hypothesis is supported by the analysis of the gut microbiota. Salmonella infection alters both alpha and beta diversity and many bacterial taxa are differentially abundant between the HS and LS. Furthermore, functional enrichment analysis reveals that strong differences between the three shedding classes are observed on day 2, 3, and 17 postinfection. Modification in bacterial taxa and functions strongly supports the idea that many bacteria in HS use anaerobic respiration rather than fermentation, which is favored in LS. This could of course be related to the utilization and adaption to the inflammatory response detected in the HS. Again, this would be clearly in line with the hypotheses put forward in mouse model studies.

## MATERIALS AND METHODS

### Experimental infection: study design and animals.

The experiment was performed at Anses BSL3 facilities, which have an agreement for animal experimentation delivered by the Direction Départementale de la Protection des Populations des Côtes d’Armor (Departmental Directorate for Protection of the Population; Anses registration no. C-22-745-1). The *in vivo* trial protocol was approved by the Ethics Committee on Animal Research no. 16, of the French Ministry of Research (APAFiS license no. 16570-2018083015249240). Anses, Inrae and University of Surrey approved the design of the animal experiment.

The trial was conducted using 45 specific-pathogen-free (SPF) Large White piglets born at Anses Ploufragan’s protected animal facilities. The piglets were weaned when they were 4 weeks old and were distributed into 5 units: one unit with 5 piglets in the same pen (control group), and four units divided in two pens, each with 10 piglets (5 per pen). Those 40 pigs represent the inoculated group. The 45 piglets were born from 5 sows and in each pen, piglets were distributed to avoid a maternal effect between the pens. Moreover, piglets were distributed following a random-cluster criteria balancing by sex and weight.

Seven days before inoculation with Salmonella, the 45 piglets were tested and confirmed to be free of Salmonella. Fecal samples from each piglet were analyzed in accordance with the NF U47-102 method (55). On day 0, at 7 weeks age, the 40 piglets were inoculated with a monophasic variant of Salmonella Typhimurium strain, as previously described (56). Briefly, piglets were orally inoculated with 10 mL of solution containing a bacterial solution of 10^8^ CFU per mL in tryptone salt broth (TSB) (bioMérieux) using a cannula connected to a screw syringe. The five control piglets received 10 mL of sterile TSB broth.

### Monitoring and sample collection during experimental infection.

During the experimental trial, rectal temperatures and clinical signs were monitored daily, whereas weight and food intake were recorded weekly. Rectal temperatures over 40.0°C indicated a pyrexia.

Three tubes of blood were collected at the jugular vein before infection at day 20 and 7 before inoculation (dbi) and then at 0, 1, 3, 7, 10, 14, 17, and 21 days postinoculation (dpi). One EDTA-stabilized tube was intended to measure total blood cells directly after sampling; another tube to determine anti-Salmonella antibody levels and cytokines in serum. These tubes were centrifuged at 2300 × *g* for 5 min and sera were stored at −20°C until analyses.

Blood was also sampled in a third EDTA-stabilized tube for gene expression analysis. Blood was immediately suspended in RNA later (Qiagen) and stored at −80°C until tested.

Feces (at least 30 g) were collected *per rectum* 7 days before inoculation and at day 1, 2, 3, 10, 14, 17, and 21 dpi for the inoculated groups and 7, 14, and 21 dpi for the control group for Salmonella detection and enumeration.

At 21 dpi, piglets were euthanized by intravenous injection with an overdose of tiletamine and zolazepam (Zoletil 100). The subsequent necropsies primarily examined organs and tissues in the abdominal and thoracic cavities. The tonsils, mesenteric lymph nodes (MLNs), and intestinal contents of the ileum and cecum were collected for Salmonella detection and enumeration.

The fecal samples and the intestinal contents of the ileum and cecum taken during the postmortem examination were immediately stored and transported on ice to the laboratory where 1 g of each sample was rapidly recovered in a cryotube and stored at −80°C until used for fecal gut microbiota analysis. Enumeration and detection of Salmonella was performed on 25 g of each sample.

### Enumeration and detection of Salmonella during experimental infection.

Enumeration of Salmonella in samples were performed as previously described by Cevallos et al. (56). If enumerations for Salmonella spp. were negative (below the enumeration limit; 10 CFU/g), the NF U47-102 method was used to confirm the presence or absence of Salmonella in the sample. Absence of Salmonella excretion for control piglets was checked throughout the assay.

To evaluate total Salmonella shedding for each pig over the course of the study, the CFU/g in feces from each sampling point was log-normalized and plotted to calculate the area under the log curve (AULC) (19, 26). Then, a hierarchical clustering (function hcclust) was performed on AULC data to cluster pigs in three classes according to their level of Salmonella shedding (high, intermediate, and low shedders, respectively, named HS, IS, and LS). A variance analysis (function lm) and a Newman and Keuls test (function SNK.test, library agricolae), were performed to confirm that these three classes were significantly different.

For the data on level of Salmonella obtained from necropsy, we compared the contamination levels of each group at the two dates of necropsy with a Kruskal-Wallis test (*P* < 0.05). The Salmonella shedding levels at each sampling day after infection were also compared using the Kruskal-Wallis test (*P* < 0.05). All the statistical analyses were performed using R software (R version 4.0.4).

### Evaluation of the immune response.

**(i) Total blood cell counts.** Blood cell counts were performed directly after sampling from EDTA blood samples using an MS9.5 hematology analyzer (Melet Schloesing Laboratoires). Thirty parameters per sample were obtained (see Table S1 in the supplemental material).

10.1128/msystems.00852-22.1 TABLE S1 Parameters measured with MS9.5 haematology analyser. Download Table S1, DOCX file, 0.02 MB. Copyright © 2023 Kempf et al. 2023 Kempf et al. https://creativecommons.org/licenses/by/4.0/ This content is distributed under the terms of the Creative Commons Attribution 4.0 International license.

**(ii) Level of Salmonella antibodies.** For antibody screening, an IDEXX Swine Salmonella *Ab* test (IDEXX) was used following the manufacturer protocol. The latter test has a sensitivity of 99.1% and a specificity of 99.4%. The presence or absence of antibodies to Salmonella in the sample was determined by calculating the S/P ratio corresponding to the absorbance value at 650 nm of the sample (S) over the mean absorbance value of the positive control (P). The results were expressed as a percentage of optical density (OD%). Seroconversion was defined as previously described by Cevalos et al. in 2019 (57) and according to the manufacturer’s recommendations.

**(iii) Level of cytokines in serum.** Porcine cytokines were quantified using the Elisa kit from Bio-techne for TNF-α, IL-1β, and IL-6, (Bio-techne R&D system) and using the Elisa kit from Invitrogen for and IFN-γ (FisherScientific). For these 3 sets of data, statistical analyses of results were performed with R software (R version 4.0.4).

**(iv) RNA extraction for transcriptomic immune response studies.** After thawing, 600 μL of each blood sample was centrifuged, the supernatant was discarded and the NucleoSpin RNA kit (Macherey-Nagel) was used to purify the RNA as described by the manufacturer. The RNA concentration was measured using the ND-1000 Nanodrop spectrophotometer. Two hundred ng of total RNA, 0.25 μg of Oligo-d(T_20_) (Eurogentec), and 0.25 μg of random primer (Promega) were denatured at 75°C for 5 min then incubated on ice for 5 min. The reverse transcription reaction was carried out in a final volume of 25 μL containing 1 mM dNTP, 30 U/μg of RNA of AMV reverse transcriptase (Promega), and 1 U/μL of RNasin (Promega), at 42°C for 60 min, then at 94°C for 5 min. After reverse transcription, cDNAs were purified using the QIAquick PCR purification kit (Qiagen). cDNA yield was measured using the ND-1000 Nanodrop spectrophotometer, and concentrations were adjusted to 50 ng/μL.

**(v) Quantitative RT PCR and transcriptomic immune response analysis.** Seventy primer pairs were used in the study to compare immune gene expression in HS and LS pigs; their sequences are indicated in Table S2. BioMark, a high-throughput PCR device from Fluidigm, was used to perform the qPCR according to manufacturer’s recommendations. A total of 63 ng of purified cDNA was amplified using the pooled primers to a final concentration of 50 nM and a thermal cycling consisting of 5 min at 95°C, followed by 18 cycles of 15 s at 95°C, and 4 min at 60°C, and a final holding step at 4°C. Thereafter, the protocol recommended by the supplier was followed (Fluidigm quick references PN 100 to 5875 B1, and PN 100 to 9791 B1). The software Fluidigm real-time PCR analysis was used to determine the Cq values of each sample/primer pair couple. The Cq were determined by the auto detector method with a quality control of 0.65 and a linear baseline correction. The fold change (FC) in gene expression between HS and LS or between control and infected pigs was calculated by the 2^-ΔΔCq^ method (58). An unpaired *t* test was used to determine the statistically significantly differentially expressed (DE) genes. The fold changes (FC) greater than 2 and less than −2, with a *P* value < 0.05 were considered for further analyses.

10.1128/msystems.00852-22.2 TABLE S2 Primer sequence used to study expression of 70 genes by qRT PCR. Primer pairs with an * were designed and produced by Fluidigm. Download Table S2, DOCX file, 0.03 MB. Copyright © 2023 Kempf et al. 2023 Kempf et al. https://creativecommons.org/licenses/by/4.0/ This content is distributed under the terms of the Creative Commons Attribution 4.0 International license.

### 16S rRNA microbial profiling using next-generation sequencing.

**(i) Sample size and metadata collection.** In total, 458 samples were collected, including fecal (*n* = 369) and intestinal tract samples (cecum, *n* = 45 and ileum, *n* = 44).

Metadata consisted of animal ID, animal dam ID, sex, sampling dates before and after inoculation, sample type (feces, ileum, cecum contents), Salmonella level (log_10_CFU/g), and shedding classes (LS, IS, HS) of each pig.

All samples were collected in sterile centrifuge tubes containing 50% glycerol and stored on cool packs until moved to −80°C. Samples remained frozen until the time of DNA extraction.

**(ii) DNA extraction and 16S sequencing.** Total DNA was extracted using the DNeasy PowerSoil kit (Qiagen) following the manufacturer’s instructions, and 16S (V3-V4 region) amplification was performed using the following primers Illumina_16S_341F (5′-TCGTCGGCAGCGTCAGATGTGTATAAGAGACAGCCTACGGGNGGCWGCAG) and Illumina_16S_805R (5′-GTCTCGTGGGCTCGGAGATGTGTATAAGAGACAGGACTACHVGGGTATCTAATC) as described by Zheng et al. in 2015 (59). 16S rRNA amplicons were sent to the Earlham Institute (UK) where sequencing was performed using an Illumina MiSeq sequencer obtaining 300-bp paired-end raw reads.

**(iii) Pig gut microbiota analysis using Qiime pipeline.** Raw sequences were analyzed using Qiime2 (version 2019.10.0) software (60, 61). Briefly, raw sequences were demultiplexed (Demux Qiime tool), trimmed and low-quality sequences were discarded (Dada2 Qiime tool option –p-trim-left 15 –p-trunc-len 220 (62). These sequences were used to generate a MAFFT tree (63) and used to construct a phylogeny with Fasttree2 (64) that was referenced against the Greengene database (65) to obtain operational taxonomic units (OTUs) that can be clustered according to the available metadata. OTU composition was analyzed using a variety of different statistical analyses and data visualization available within Qiime2 software: Alpha rarefaction (shows the OTUs number/sequencing depth); Alpha distribution (OTUs diversity within each group); Beta distribution (OTUs diversity between each group); and OTUs boxplot and principal component analysis (PCA), which can be calculated considering the presence/absence of OTUs Jaccard) or considering the OTUs relative abundance (UniFrac based). OTU tables were also imported in Orange3 statistical software (66) to explore different statistical analyses to test for associations between Salmonella shedding status and changes in the gut microbiota such as K-means and MDS. Metadata were validated to be suitable for the use in Qiime2 by using Keemei Google Docs plug-in (67).

**(iv) Pig gut microbiota analysis using FROGS pipeline.** Data were uploaded on FROGS analysis pipeline (32) which was used for all further steps of gut microbiota characterization. First, paired-end reads from each sample were clustered by allowing a mismatch rate of 0.1. They were next selected using an expected read size of 300 bp, and a total amplicon size ranging from 400 bp to 600 bp with a mean of 460 bp. The resulting sequences were clustered using Swarm (68). For this, we used 1 and 1 as the values of aggregation distance parameters (for the denoising and final clustering steps, respectively). OTUs, including chimeric sequences, were then removed using VSearch (69). Other quality control steps included removal of very rare OTUs (relative abundance < 0.0005% of the total read numbers) and those including sequences matching phiX sequences recorded in a specific data bank (32). Finally, the resulting OTUs were classified using an NCBI BLAST+ search within the Silva SSU 123 database (70, 71).

Diversity assessment was based on the Chao1, Shannon α-diversity indexes and the Bray-Curtis β-diversity index. Alpha diversity within each shedding category were compared using one-way ANOVA, computed using the dedicated native function of R environment (72). Comparison of β-diversity indexes were conducted using PERMANOVA tests performed using the adonis() function of the R-package Vegan (73); bacterial counts were fitted onto the β-diversity ordination using the envfit() function of the R-package Vegan. Differential abundances were assessed following the hypothesis that abundances in each sample followed negative binomial distributions. Under this scheme, the abundance may be modeled by fitting a generalized linear model. Significant logarithmic fold change ratios were detected using Wald tests and Benjamini-Hochberg adjustment for multiple testing (*P* < 0.01). The computations were performed using the R-package DESeq2 (74).

**(v) Functional metagenomic predictions based on pig gut microbiota analysis.** Functional gene families and MetaCyc pathways were predicted using the PICRUST2 package (75). MetaCyc pathways were aggregated at the superpathway level using the MetaCyc database (76).
