# Supplementary material for: Inflammatory Responses Induced by the Monophasic Variant of Salmonella Typhimurium in Pigs Play a Role in the High Shedder Phenotype and Fecal Microbiota Composition
Source: mSystems. 2023 Jan 11;8(1):e00852-22. doi: 10.1128/msystems.00852-22 (PMC9948705; doi:10.1128/msystems.00852-22)
Supplement: TABLE S1 [file msystems.00852-22-s0001.docx]

**S1 Table. Parameters measured with MS9.5 haematology analyser**

| **Mesured parameters** | **description** | **Value expression** |
| --- | --- | --- |
| **GB** | Whites cells (leucocytes) | m/mm3 |
| **% lympho** | Percentage of lymphocytes | % |
| **% mono** | Percentage of monocytes | % |
| **% N/Gr** | Percentage of granulocytes | % |
| **% eosino** | Percentage of éosinophiles | % |
| **% baso** | Percentage of basophiles | % |
| **% autre** | Percentage of other cells | % |
| **# lymph** | Lymphocytes count | # |
| **# mono** | Monocytes count | # |
| **# N/Gr** | Granulocytes count | # |
| **# eosino** | Eosinophils count | # |
| **# baso** | Basophiles count | # |
| **# Autre** | Other cells count | # |
| **GR** | Red Cells | m/mm3 |
| **VGM** | Average volume of blood cell (Hct/RBC) | fl |
| **Hct** | Haematocrit | % (ou l/l) |
| **TCMH** | Mean corpuscular haemoglobin concentration | pg |
| **CCMH** | Mean cell haemoglobin concentration | g/dl |
| **Hbg** | Haemoglobin | g/dl |
| **IDR-SD ; IDR-CV** | Red blood cell distribution index | fl |
| **µGR** | Micro-red cells count | % |
| **MGR** | Macro-globules-cells count | % |
| **Plt** | Platelets | m/mm3 |
| **VMP** | Average platelet volume | fl* |
| **Pct** | Plateletcrite | % |
| **Mode** | Platelet mode | fl |
| **Medn** | Platelet median | fl |
| **IDP** | Platelet distribution index |  |
| **µplt** | Micro-platelets count | % |
| **MPlt** | Macro-platelets count | % |
| fl* = femtolitres |  |  |
