# Supplementary material for: Inflammatory Responses Induced by the Monophasic Variant of Salmonella Typhimurium in Pigs Play a Role in the High Shedder Phenotype and Fecal Microbiota Composition
Source: mSystems. 2023 Jan 11;8(1):e00852-22. doi: 10.1128/msystems.00852-22 (PMC9948705; doi:10.1128/msystems.00852-22)
Supplement: TABLE S2 [file msystems.00852-22-s0002.docx]

**S2 Table. Primer sequence used to study expression of 70 genes by qRT PCR.** Primer pairs with an * were designed and produced by Fluidigm.

| **Gene** | **Forward primer** | **Reverse primer** |
| --- | --- | --- |
| *ALOX5AP** | AGGAAGCCATGGGCAACA | TCCACCTTGTGGGCAAAGAA |
| *ARG1** | ATCATCGGAGCTCCTTTCTCC | AGCAGACCAGCCTTTCTCAA |
| *ARG2** | TCATTGGAGCCCCATTCTCA | TCAAGCCAGCTTCCCTTACA |
| *ARPC4** | GAACGACACAACAAGCCAGAA | TCATTCCTGCTGATGGTCACA |
| *B2M** | CGAGACCACTAACCGGCATCA | TGGATTCATCCAACCCAGATGCA |
| *CASP1* | AACTCTCCACAGGTTCACAAT | TGGGTAAATCTCTGCTGACT |
| *CASP4* | CAGAGGTGCAAATCGTGGAGAAG | GTGACAGCGCCAAGCATATTT |
| *CASP8* | AGATGTCCCAGGGGTGAAGA | GGCACTACCCCTTCAATCTA |
| *CD14** | AGTACGCGGACATGCTCAA | GGCCAGAATCTGAGCAGGAA |
| *CD80* | TTAATGGTCAAAGCTGACTTTC | ATTTTCCAACCAGGAGAGGT |
| *CD81* | ATCGCTGTGGGTGCTGTGAT | TTGAGCGTCTCGTGGAAAGT |
| *CD86** | ATGAACAGGAAGGCGAGTGAA | ACATCACACTGGGCATCATCA |
| *CEBPB** | CGCAGGTCAAGAGTAAGACCAA | GCTCACGCCGGATCTTGTA |
| *CLEC7A* | GAGTTCTTTCTGGCTCTTGT | TCTGGCTGTGAGAAAGCCCT |
| *CSF3** | CAGCTGGATGTCACCGACTTA | GGCCATCCTCAGGTCTTCC |
| *DDX3X** | AACACCACTACAAGGGTAGCA | TAGTCTCTGGCACCGAATCC |
| *EIF2AK2* | CTGCACATAACTTGAGGTTTAC | GCCTATGTAATTCTCAGTGAG |
| *GAPDH** | TGTCTTCACGACCATGGAGAA | GTTCACGCCCATCACAAACA |
| *GATA3* | GGCGAGGTCCAGCACAGAAG | TGCACAGGACGTACCTGCCCT |
| *GNLY* | ACCACAGCCCAACGAGGACAC | CCACCTGTCTTCTCTTTACAG |
| *HSP60* | CCCCGCAGAAATGCTTCGAT | GACTGCCCCAACTCTGTTCA |
| *HSP70* | TTCCGAAAGCTGGTGCATCT | TTGCTAGGATCTCCACCCGA |
| *HSP90* | AGAAAGTCGAAAAGGTGGTTG | GCCTCTGCCTTTTGCCTCAG |
| *IDO* | AGCCCTTGAAGTGTTTCACCA | CTGAAGGAACTCCACCCACA |
| *IFNA* | GACTTTGGATCCCCTCATGA | TGTGCTGAAGAGCTGGAAGG |
| *IFNAR1* | CGTTCGCCAAGAAAAGTCGG | CTTGTCTGAATGGTCCTCAGG |
| *IFNAR2* | AGGGTCCTTGATGACGACGAT | GTCTCAGTGGCATCCTCTAGC |
| *IFNGR1** | ACTCTGGAGCCCGGTGTTTA | GGTATTGCAGGCATCGATCCA |
| *IGSF6** | CCAGTTCGAAGGAGCCAAAA | TGCAGTTCCTTGCTGAGAAC |
| *IL1* | TGTTCAGGTTTCTGAAGCAGC | TTCTCCACTGCCACGATGAC |
| *IL2* | GCTGATCTCTCCAGGATGCTC | ACTTGTTTCAGATCCCTTTAGT |
| *IL4** | TGAGCGGACTTGACAGGAA | GCTCTTCTTGGCTTCATGCA |
| *IL10* | GCGGCGCTGTCATCAATTTC | TGGCTTTGTAGACACCCCTC |
| *IL15* | ATTTTGGGCTGTATCAGTGCAG | GCAAAATGACGCGTAACTCCA |
| *IL18* | GCTGCTGAACCGGAAGACAA | CCGATTCCAGGTCTTCATCGT |
| *IRF2* | ACGGTGAACATCATAGGACAGT | ATCACTGGGCACACTATCGG |
| *IL23A** | ACTGGGAGACTGAGCAGAC | AAGGATCTTGAGGCGGAGAA |
| *LEF1** | ACCATGACAAGGCCAGAGAA | GTAAGAGGGCCCCTTGTTGTA |
| *MDA5* | AGCCACAGATCAGCCAAGTC | AGCCACAGTCTCTTCATCTGAATC |
| *MX1* | GGAGAGAGTTGCCAGGGTTT | TTTTGGACTTGGCAGTTCTGTG |
| *MX2* | GGGAAATACGCAAAGCCCAG | TCTGCTGCTCCTGGATGTAC |
| *MYD88* | CCATTCGAGATGACCCCCTG | ATCCGACGGCACCTCTTTTC |
| *NFKB1* | AAGGCAGCAAATAGACGAGC | GTGGGGCACTTTGTTGAGAG |
| *NLRP3* | AAGAGGGATGAGCCAGAATG | ACTCTCACCCAGACGTGCAT |
| *PGK1** | GAAGAACAACCAGATAACGAACAAC | TGACTTGGCTCCGTTGTCTA |
| *RELA** | AATCAGCGCATCCAGACCAA | ACAGCATTCAGGTCGTAGTCC |
| *S100A12** | AGCAGCTGATCACCAAGGAA | GATTGGCATCCAGGTTTTGGAA |
| *S100A9** | CGCTCCTTGCTGTCCAAC | CATTTGGTCCGCCATCTTCC |
| *SDCBP** | TGCGAGGTCAATGGACAGAA | GATGACATTCCCAGCTGTTGAC |
| *SLA1* | CATCATTGTTGGCCTGGTTC | TCCTTTTTCACCTGAGCGCT |
| *SLADMB* | GTTCCTGAGCCCATCCTTCA | GAAAATGTAGCCTGGGGCAA |
| *SLADQB* | CGTTGTGTCCACTCCTCTTA | ACGAGCCCCTTCTGACTCCT |
| *SLADRB* | GTTCCTCAGAGTGGAGAGGT | GCGTCCTTTCTGATTCTTGAAG |
| *SOCS1* | CTATGGCCCACCCCTCCG | CTCTGCTGCCGTGGAGATTG |
| *SOCS3* | CTTCACGCTCAGCGTCAAG | CTTGAGCACGCAGTCGAAG |
| *STAT4* | ACCATTCGCTGACATCCTTC | CAGTTGAATCACTTCGGATTG |
| *STAT6* | CTCAGCCCAGATATGGTGTCC | TGAGGTTGGTCAAAGGGCAG |
| *TBX21* | AAGTGGGTGCAGTGTGGAAA | AGCACGATCATCTGCGTCAT |
| *TGFBR1* | TGGACTTGCCCATCTTCACA | TCAGGGGCCATGTACCTTTT |
| *TGFBR3* | AAATTGGGCCCGAAAGGAGT | ACTGTTGGGAGCGAGAACTT |
| *TLR1** | GCCAGCAGGACAGAGAAAC | TCCAACACAGAGGAGAGTCC |
| *TLR2* | TTAAAGACGGTGTGCTGCAAG | CAGCAGGGTCACAAGACAGA |
| *TLR3* | AAATGAATCACCCTGCCTAGC | ACAAGGCAAACTCCTGCTCA |
| *TLR4** | TGGTGTCCCAGCACTTCATA | CGGCATGACTCCTCAGAAAC |
| *TLR5** | CCAGTACAGCGACCAAAACA | TGTTGCTGATGCGATCTTCC |
| *TLR6* | TCTCATGGCACAGCGAACTT | CACATCATCCTCTTCAGCGACT |
| *TLR8** | CCCGCAACTTGGTTTGAGAA | GAGGCGATTTCGTCCATCAAA |
| *TNFRSF1A** | TTAGCTTGCCGCTACCAAC | CCTTTACAGGAGTCGATTTCCC |
| *TNFSF5* | AAATGCACAAAGGCGATCAGG | GTGTAGTACCCTTTGGGGGC |
| *TREM1** | TGGCCTCAAAGTTACCCTCA | ACACACCGCAGGAAGGATA |
